# Effects of Prior Local Therapy by Radical Prostatectomy or Radiotherapy on the Efficacy and Quality of Life of Patients Treated With Darolutamide in ARAMIS

**DOI:** 10.1002/cam4.71343

**Published:** 2025-12-26

**Authors:** Matthias Saar, Karim Fizazi, Neal D. Shore, Matthew Smith, Jan‐Erik Damber, Andrey Semenov, Maria J. Ribal, Alison Birtle, Jérôme Rigaud, Christopher J. D. Wallis, Marc‐Oliver Grimm, Susan Halabi, Andrew J. Armstrong, Ateesha F. Mohamed, Patrick Adorjan, Shankar Srinivasan, Frank Verholen, Alicia K. Morgans, D. Robert Siemens

**Affiliations:** ^1^ Department of Urology and Pediatric Urology University Medical Center RWTH Aachen Aachen Germany; ^2^ Institut Gustave Roussy University of Paris Saclay Villejuif France; ^3^ Carolina Urologic Research Center Myrtle Beach South Carolina USA; ^4^ Massachusetts General Hospital Boston Massachusetts USA; ^5^ University of Gothenburg Gothenburg Sweden; ^6^ Ivanovo Regional Oncology Dispensary Ivanovo Russia; ^7^ Uro‐Oncology Unit, Hospital Clinic University of Barcelona Barcelona Spain; ^8^ Rosemere Cancer Centre Lancashire Teaching Hospitals Preston UK; ^9^ University of Manchester Manchester UK; ^10^ University of Central Lancashire Preston UK; ^11^ Nantes University Hospital Centre Nantes France; ^12^ Division of Urology, Department of Surgery University of Toronto Toronto Ontario Canada; ^13^ Mount Sinai Hospital Toronto Ontario Canada; ^14^ University Health Network Toronto Ontario Canada; ^15^ Jena University Hospital Jena Germany; ^16^ Duke University Division of Biostatistics Durham North Carolina USA; ^17^ Duke Cancer Institute Center for Prostate and Urologic Cancers, Duke University Department of Medicine, Division of Medical Oncology Durham North Carolina USA; ^18^ Bayer HealthCare Pharmaceuticals Inc. Whippany New Jersey USA; ^19^ Bayer Consumer Care AG Basel Switzerland; ^20^ Dana‐Farber Cancer Institute Boston MA USA; ^21^ Queen's University Kingston Ontario Canada

**Keywords:** androgen receptor inhibitor, darolutamide, metastatic‐free survival, nonmetastatic, castration‐resistant prostate cancer, overall survival, prostatectomy, radiotherapy

## Abstract

**Background:**

Darolutamide plus androgen‐deprivation therapy (ADT) improved metastasis‐free survival (MFS) by 2 years and reduced the risk of death by 31% in nonmetastatic castration‐resistant prostate cancer (nmCRPC) in ARAMIS. Prior local therapy may influence the efficacy of subsequent systemic therapy. This post hoc analysis of ARAMIS evaluated the effect of prior local therapy on the efficacy and health‐related quality of life (HRQoL) of darolutamide.

**Methods:**

Patients with nmCRPC were randomized to darolutamide (*n* = 955) or placebo (*n* = 554) while continuing ADT. MFS, overall survival (OS), time to prostate‐specific antigen (PSA) progression, and HRQoL deterioration‐free survival (DetFS) were estimated for patients with and without local therapy and by treatment using Kaplan–Meier methods.

**Results:**

Darolutamide increased MFS versus placebo in patients with (HR, 0.36; 95% CI, 0.26–0.48) and without (HR, 0.46; 95% CI, 0.36–0.59) local therapy. Median OS was 48.6 months for placebo without local therapy and not reached in either the darolutamide group or placebo group with local therapy. Darolutamide 3‐year OS rates were 86.9% (95% CI, 83.0–90.8) and 79.0% (95% CI, 66.2–78.1) in patients with and without local therapy, respectively. Darolutamide showed evidence of improved OS versus placebo in patients with prior local therapy (HR, 0.80; 95% CI, 0.50–1.30) and a greater effect in those without local therapy (HR, 0.67; 95% CI, 0.50–0.90). Darolutamide delayed time to PSA progression and HRQoL deterioration regardless of local therapy.

**Conclusions:**

Darolutamide versus placebo improved MFS, OS, time to PSA progression, and HRQoL DetFS independent of prior local therapy, consistent with the overall ARAMIS population.

**Trial Registration:** ClinicalTrials.gov registration: NCT02200614

## 1 Introduction

The primary treatment for localized prostate cancer is radical prostatectomy (RP) and/or radiotherapy (RT), thereby reducing the risk of distant metastases [1, 2]. Historically, when patients experienced disease relapse, a majority of patients would start at least androgen‐deprivation therapy (ADT) [3, 4]. While ADT can effectively reduce prostate‐specific antigen (PSA) levels and control progression for a period of time, many patients progress with the development of castration‐resistant disease [1, 5]. Patients with nonmetastatic, castration‐resistant prostate cancer (nmCRPC) have no detectable metastases on conventional imaging and rising levels of PSA [1, 5]. The key therapeutic goal for most patients with nmCRPC is to delay metastases and reduce subsequent morbidity and mortality with well‐tolerated therapeutics [5]. A portion of patients are diagnosed with nmCRPC following hormonal therapy for locally advanced disease without receiving a primary local therapy.

Darolutamide is structurally different from other androgen‐receptor inhibitors (ARIs) [6]. Treatment with darolutamide reduces disease progression or death with a low rate of adverse events (AEs) in both the nmCRPC and metastatic hormone‐sensitive prostate cancer settings [7, 8, 9, 10, 11]. The double‐blind, placebo‐controlled, phase 3 ARAMIS trial demonstrated that treatment with darolutamide and ADT led to a statistically significant increase in median metastasis‐free survival (MFS) by nearly 2 years and reduced the risk of death by 31% (hazard ratio [HR], 0.69; 95% confidence interval [CI], 0.53–0.88; *p* = 0.003) versus ADT alone in patients with nmCRPC [7, 8]. Discontinuation rates due to AEs among patients treated with darolutamide remained consistently low and similar to placebo (8.9% vs. 8.7%; respectively) after extended follow‐up [7, 8].

The impact of prior local therapy on the survival and prognosis of patients with nmCRPC has not been well defined [12, 13]. Two small studies demonstrated potentially improved survival among patients who underwent RP prior to developing CRPC [12, 14]. Local therapy may improve survival for a number of reasons, including a reduction of tumor volume, inhibition of cellular dissemination from the primary tumor, and inhibition of tumor‐promoting signaling from the primary tumor [14].

Despite previous results on the outcome of patients with nmCRPC who had received prior local therapy [13], evidence of differential outcome on overall survival (OS) according to the presence or absence of prior local therapy is still required. Due to the gap of evidence around the effect of prior local therapy, this post hoc analysis of ARAMIS evaluated the efficacy of darolutamide in patients with or without prior local therapy of RP or RT to the primary tumor. We also evaluated the effect of darolutamide on health‐related quality of life (HRQoL) deterioration‐free survival (DetFS) and the safety profile of darolutamide by prior local therapy.

## 2 Methods

### 2.1 Trial Design and Participants

ARAMIS (NCT02200614) was a phase 3, randomized, double‐blind, placebo‐controlled trial that compared darolutamide plus ADT with ADT alone in patients with nmCRPC. The methods have been reported previously [7] and are summarized here. The institutional review board at each center (409 centers in 36 countries) approved the trial, which was conducted in compliance with the principles of the Declaration of Helsinki and in accordance with the International Conference on Harmonization guidelines for Good Clinical Practice.

Patients were eligible if they were ≥ 18 years old with histologically or cytologically confirmed adenocarcinoma of the prostate. Patients were required to have castration‐resistant prostate cancer, a baseline PSA level of ≥ 2 ng/mL, a PSA doubling time of ≤ 10 months, and an Eastern Cooperative Oncology Group (ECOG) performance status of 0 or 1. All patients underwent radionuclide bone scanning, computed tomography, or magnetic resonance imaging for metastases screening. Patients with detectable metastases or a history of metastatic disease on conventional imaging were excluded, apart from those with pelvic lymph nodes < 2 cm in diameter in the short axis below the aortic bifurcation.

Patients were enrolled from September 2014 through March 2018 and the data cutoff for the final analysis was November 15, 2019. Patients were randomized 2:1 to receive either darolutamide 600 mg twice daily or matched placebo during the double‐blind treatment period and continued receiving ADT. Randomization was stratified according to PSA doubling time (≤ 6 months or > 6 months) and the use of osteoclast‐targeted therapy at randomization (yes or no). During the open‐label period, patients in the darolutamide treatment group continued darolutamide and patients previously receiving placebo were allowed to cross over to receive darolutamide or other life‐prolonging therapy.

### 2.2 Assessments

Demographic characteristics and relevant medical history were obtained at screening. Disease assessments, AEs, vital signs, and laboratory safety assessments were collected at each visit (every 16 weeks). Efficacy was evaluated in all randomized patients in the intention‐to‐treat population. MFS analysis was performed after metastasis or death had occurred in 437 patients at the primary completion date (September 3, 2018) [7]. All other efficacy analyses used the final analysis data cutoff date (November 15, 2019). Safety was evaluated in patients who received ≥ 1 dose of darolutamide or placebo during the double‐blind treatment period.

In this post hoc exploratory analysis, MFS, OS, time to PSA progression, PSA response, and safety were assessed for patients who received prior local therapy (RP or RT) versus those who did not and by treatment group. MFS was defined as the time from randomization to confirmed evidence of distant metastasis on conventional imaging or death from any cause, whichever occurred first. PSA progression was defined as an increase of PSA of ≥ 25% and an absolute increase of PSA of ≥ 2 ng/mL above the nadir, which was confirmed by a consecutive value obtained ≥ 3 weeks later, and based on the Prostate Cancer Working Group 2 (PCWG2) criteria [15]. PSA response was defined as ≥ 50% decline from baseline (PSA50).

DetFS was defined as the time from randomization until the earliest event of deterioration in HRQoL measures using the European Organisation for Research and Treatment of Cancer Quality of Life Questionnaire‐Prostate Cancer Module (EORTC QLQ‐PR 25) urinary and bowel symptoms subscales [16] and the Functional Assessment of Cancer Therapy‐Prostate prostate cancer subscale (FACT‐P PCS) [17], metastasis, death or treatment discontinuation. Deterioration in HRQoL measures was the first decline from baseline ≥ minimally important difference, defined as the upward round off of half the standard deviation of the baseline value for each item [18].

### 2.3 Statistical Analyses

For MFS, OS, time to PSA progression, and DetFS, medians and 95% CIs were computed using Kaplan–Meier curves. The HRs and 95% CIs for treatment comparisons were estimated using the Cox regression method, and for MFS, OS and time to PSA progression, adjusted for differences between those who had prior local therapy (RP or RT) and those who did not for baseline characteristics of age, ECOG performance status, Gleason score, and time to diagnosis, which were different across groups. PSA variables were not included because they might define the groups, particularly those who received RP as they would produce less PSA. Interaction tests were conducted for MFS, OS, and time to PSA progression between study treatment groups and prior local therapy. DetFS analyses were unadjusted for baseline covariates due to the low sample size for subgroup analysis. PSA50 response rates and AEs were summarized across the prior therapy subgroups for the darolutamide and placebo groups.

## 3 Results

### 3.1 Patients

Of 1509 patients with nmCRPC randomized in ARAMIS, 954 received darolutamide and 554 received placebo [7]. Among the 954 patients treated with darolutamide, 239 and 177 received prior RP and RT, respectively, and 134 and 89 patients in the placebo group received prior RP and RT, respectively. Baseline demographics and patient characteristics were generally similar between prior local therapy subgroups and the overall population of patients treated with darolutamide (Table 1). Patients treated with darolutamide who did not have prior RP or RT had a shorter median time from initial diagnosis to study treatment (74.5 months; range 2.6–242.1) compared with patients who had RP or RT (107.6 months; range 14.2–337.5). A higher proportion of patients (36.2%) who did not receive RP or RT had ECOG performance status of 1 compared with those that did receive treatment (26.4%). The differences in baseline findings were similarly observed in the placebo group.

**TABLE 1:** Baseline demographics and patient characteristics by prior local therapy.

Characteristic at baseline	Darolutamide^a^				Placebo			
	RP (*n* = 239)	RT (*n* = 177)	RP or RT (*n* = 416)	Neither RP nor RT (*n* = 538)	RP (*n* = 134)	RT (*n* = 89)	RP or RT (*n* = 223)	Neither RP nor RT (*n* = 331)
Age, median (range), years	72.0 (52–88)	74.0 (56–90)	72.0 (52–90)	76.0 (48–95)	71.0 (50–92)	72.0 (55–90)	71.0 (50–92)	76.0 (52–91)
PSA, median (range), ng/mL	6.2 (1.8–132.8)	9.8 (1.7–164.4)	7.2 (1.7–164.4)	10.4 (0.3–858.3)	7.1 (2.0–885.2)	10.5 (2.3–94.3)	8.5 (2.0–885.2)	10.3 (1.5–310.2)
PSADT, median (range), months	3.8 (0.7–10.4)	4.5 (0.9–10.1)	4.1 (0.7–10.4)	4.7 (1.0–11.0)	3.7 (0.7–9.9)	4.3 (1.2–9.9)	4.0 (0.7–9.9)	5.0 (1.3–13.2)
ECOG PS, *n* (%)								
0	181 (75.7)	125 (70.6)	306 (73.6)	343 (63.8)	109 (81.3)	66 (74.2)	175 (78.5)	216 (65.3)
1	58 (24.3)	52 (29.4)	110 (26.4)	195 (36.2)	25 (18.7)	23 (25.8)	48 (21.5)	115 (34.7)
Gleason score, *n* (%)								
< 7	53 (22.2)	52 (29.4)	105 (25.2)	112 (20.8)	30 (22.4)	33 (37.1)	63 (28.3)	79 (23.9)
≥ 7	182 (76.2)	116 (65.5)	298 (71.6)	413 (76.8)	101 (75.4)	53 (59.6)	154 (69.1)	241 (72.8)
Time from initial diagnosis to study treatment, median (range), months	107.0 (14.2–337.5)	107.9 (15.7–258.1)	107.6 (14.2–337.5)	74.5 (2.6–242.1)	95.2 (7.0–344.7)	109.2 (19.3–250.3)	104.7 (7.0–344.7)	73.3 (0.5–263.7)

Abbreviations: ECOG PS, Eastern Cooperative Oncology Group performance status; PSA, prostate‐specific antigen; PSADT, prostate‐specific antigen doubling time; RP, prostatectomy; RT, radiotherapy.

^a^ One patient had no information on prior local therapy.

### 3.2 Metastasis‐Free Survival

**FIGURE 1:**
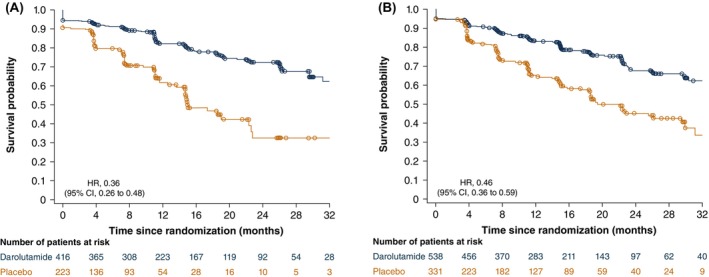
Kaplan–Meier estimates of MFS for patients with (A) and without (B) prior local therapy of RP or RT. CI, confidence interval; HR, hazard ratio; MFS, metastasis‐free survival; RP, radical prostatectomy; RT, radiotherapy.

The median MFS was consistent across prior therapy subgroups receiving darolutamide, exceeding 40 months in all groups except prior RT, where the median was not reached (Table S1). Among patients receiving placebo, the median MFS ranged between 14.7 and 19.1 months for the prior therapy subgroups. Treatment with darolutamide reduced the risk of metastasis or death among patients who received (HR, 0.36; 95% CI, 0.26–0.48) or did not receive (HR, 0.46; 95% CI, 0.36–0.59) prior local therapy compared with placebo (Figure 1A,B). There was no interaction between study treatment and prior local therapy for MFS (interaction effect HR, 0.78; 95% CI, 0.52–1.18).

### 3.3 Overall Survival

**FIGURE 2:**
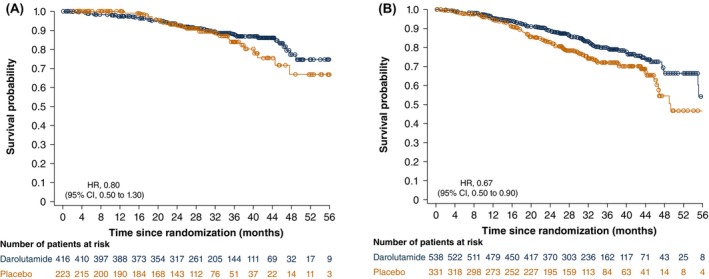
Kaplan–Meier estimates of OS for patients with (A) and without (B) prior local therapy of RP or RT. CI, confidence interval; HR, hazard ratio; OS, overall survival; RP, radical prostatectomy; RT, radiotherapy.

Across both treatment groups, patients who received prior local therapy had higher 3‐year survival rates than those who did not. The benefit of darolutamide on the 3‐year survival rate (95% CI) for patients with and without prior local therapy (86.9% [83.0–90.8] and 79.0% [74.8–83.3], respectively) was consistent with the overall darolutamide population (82.6% [79.6–85.5]; Figure S1A). In the placebo group, the 3‐year survival rates were 84.0% [77.1–90.9] and 72.1% [66.2–78.1] for those with and without prior local therapy, respectively, and were consistent with the overall placebo population (76.9% [72.4–81.4]; Figure S1B). Among patients treated with darolutamide, median OS was not reached in any prior therapy subgroup or the overall population. Median OS was lower in patients in the placebo group who did not receive prior RP or RT (median 48.6 months; 95% CI, 45.6–not estimable) and was not reached in either the darolutamide group or the placebo group who received prior RP or RT. Treatment with darolutamide versus placebo showed evidence of improved survival in patients who received prior RP or RT (HR, 0.80; 95% CI, 0.50–1.30; Figure 2A) but there was a more pronounced 33% reduction in the risk of death for patients who did not receive RP or RT (HR, 0.67; 95% CI, 0.50–0.90; Figure 2B). There was no interaction between study treatment and prior local therapy for OS (interaction effect HR, 1.19; 95% CI, 0.69–2.08).

### 3.4 Time to PSA Progression

**FIGURE 3:**
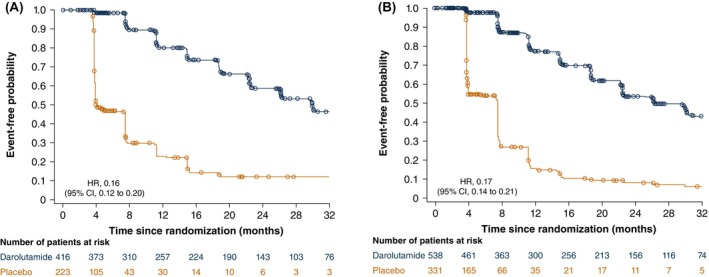
Kaplan–Meier estimates of time to PSA progression for patients with (A) and without (B) prior local therapy of RP or RT. CI, confidence interval; HR, hazard ratio; PSA, prostate‐specific antigen; RP, radical prostatectomy; RT, radiotherapy.

The median time to PSA progression was not different in patients treated with darolutamide with and without prior therapy (29.5 [95% CI, 25.9–33.3] and 26.0 [22.2–29.9] months) and was consistent with the overall darolutamide group in ARAMIS (29.5 [25.8–29.7] months). For patients receiving placebo, the median time to PSA progression was shorter at 4.0 [3.8–7.4] months for patients receiving RP or RT compared with 7.4 [3.9–7.4] months for those who did not receive prior local therapy, consistent with the overall placebo group (7.2 [3.9–7.4] months). The time to PSA progression was longer in the darolutamide versus placebo group among patients who received prior RP or RT (HR, 0.16; 95% CI, 0.12–0.20) and those who did not receive prior RP or RT (HR, 0.17; 95% CI, 0.14–0.21; Figure 3A,B). There was no interaction between study treatment and prior local therapy for time to PSA progression (interaction effect HR, 0.94, 95% CI, 0.67–1.33).

### 3.5 PSA Response Rates

PSA50 response rates for patients receiving darolutamide were similar across the prior local therapy subgroups (range, 83.8%–85.8%) and similar to the overall darolutamide population (84.6%) (Figure S2A). Among the placebo group, PSA50 response rates were lower than in the darolutamide group, similar across the prior local therapy subgroups (range, 30.3%–33.6%), and consistent with the overall placebo group (33.0%; Figure S2B).

### 3.6 HRQoL Deterioration‐Free Survival

Darolutamide improved DetFS for EORTC QLQ‐PR 25 urinary symptoms by 40% regardless of prior local therapy, but median DetFS was shorter in the prior RP or RT versus no prior RP or RT group, suggesting a negative effect of local therapy on urinary symptoms (Table 2). Darolutamide also improved DetFS for EORTC QLQ‐PR 25 bowel symptoms and FACT‐P PCS, with a greater effect in the prior local therapy group compared with the no prior local therapy group (38%–41% vs. 20%–26%). HRQoL deterioration events contributed to more than 55% of total DetFS events in the darolutamide group and 40%–50% in the placebo group, while earlier metastases contributed to 25%–30% of events in the darolutamide group and 40%–50% of events in the placebo group (Table S2).

**TABLE 2:** HRQoL DetFS in patients with nmCRPC by prior local therapy.

DetFS median (95% CI), months	RP		RT		Prior RP/RT		No prior RP/RT	
	Darolutamide (*n* = 239)	Placebo (*n* = 134)	Darolutamide (*n* = 177)	Placebo (*n* = 89)	Darolutamide (*n* = 416)	Placebo (*n* = 223)	Darolutamide (*n* = 538)	Placebo (*n* = 331)
EORTC QLQ‐PR 25 urinary symptoms subscale	11.1 (11.0–15.4)	7.5 (7.3–11.1)	18.3 (11.4–22.1)	7.4 (4.9–11.1)	14.8 (11.1–18.4)	7.4 (7.3–11.0)	18.4 (14.8–21.9)	10.9 (7.4–11.1)
HR (95% CI)	0.65 (0.49–0.87)		0.51 (0.36–0.72)		0.59 (0.47–0.73)		0.60 (0.50–0.73)	
EORTC QLQ‐PR 25 bowel symptoms subscale	11.6 (10.9–18.4)	7.4 (7.2–11.1)	14.8 (10.9–18.4)	7.4 (3.8–11.1)	14.6 (11.1–18.3)	7.4 (7.3–11.0)	14.7 (11.1–14.9)	9.4 (7.4–11.1)
HR (95% CI)	0.61 (0.45–0.82)		0.57 (0.40–0.81)		0.59 (0.47–0.74)		0.74 (0.62–0.89)	
FACT‐P PCS	21.9 (11.4–40.4)	7.7 (7.2–11.3)	11.1 (7.6–18.3)	7.4 (3.8–12.4)	14.9 (11.1–18.5)	7.6 (7.3–11.1)	11.1 (11.0–14.8)	10.9 (7.4–11.1)
HR (95% CI)	0.55 (0.41–0.75)		0.71 (0.51–1.00)		0.62 (0.49–0.78)		0.80 (0.67–0.96)	

Abbreviations: DetFS, deterioration‐free survival; EORTC QLC‐PR 25, European Organisation for Research and Treatment of Cancer Quality of Life Questionnaire‐Prostate Cancer Module; FACT‐P PCS, Functional Assessment of Cancer Therapy‐Prostate Prostate Cancer Subscale; HRQoL, health‐related quality of life; RP, radical prostatectomy; RT, radiotherapy.

### 3.7 Safety

Overall, the safety profile of darolutamide was consistent for patients with and without prior local therapy and similar to the placebo group (Table 3). AEs occurred in 87.7% and 84.2% of patients receiving darolutamide and 77.6% and 80.4% of patients receiving placebo with and without RP or RT, respectively. Grade 3 or 4 AEs occurred in 26.7% and 26.0% of those receiving darolutamide and 22.9% and 20.8% of those receiving placebo with and without prior local therapy, respectively. The percentage of patients who discontinued because of AEs was similar for patients with and without RP or RT for the darolutamide (range, 7.1%–9.9%) and placebo groups (range, 7.5%–9.1%).

**TABLE 3:** Incidences of AEs in darolutamide‐treated patients during the double‐blind treatment period by prior therapy.

Adverse events, *n* (%)	Darolutamide^a^				Placebo			
	RP (*n* = 238)^b^	RT (*n* = 177)	RP or RT (*n* = 415)^b^	Neither RP nor RT (*n* = 538)	RP (*n* = 134)	RT (*n* = 89)	RP or RT (*n* = 223)	Neither RP nor RT (*n* = 331)
Any AE	206 (86.6)	158 (89.3)	364 (87.7)	453 (84.2)	101 (75.4)	72 (80.9)	173 (77.6)	266 (80.4)
Grade 3 or 4 AE	60 (25.2)	51 (28.8)	111 (26.7)	140 (26.0)	28 (20.9)	23 (25.8)	51 (22.9)	69 (20.8)
Serious AEs	50 (21.0)	52 (29.4)	102 (24.6)	147 (27.3)	16 (11.9)	25 (28.1)	41 (18.4)	80 (24.2)
AEs leading to discontinuation	17 (7.1)	15 (8.5)	32 (7.7)	53 (9.9)	10 (7.5)	8 (9.0)	18 (8.1)	30 (9.1)
AEs of interest commonly associated with androgen receptor pathway inhibitors								
Fatigue	42 (17.6)	23 (13.0)	65 (15.7)	61 (11.3)	16 (11.9)	8 (9.0)	24 (10.8)	22 (6.6)
Hypertension	26 (10.9)	14 (7.9)	40 (9.6)	34 (6.3)	7 (5.2)	7 (7.9)	14 (6.3)	22 (6.6)
Bone fracture^c^	16 (6.7)	8 (4.5)	24 (5.8)	28 (5.2)	4 (3.0)	2 (2.2)	6 (2.7)	14 (4.2)
Falls	14 (5.9)	10 (5.6)	24 (5.8)	26 (4.8)	7 (5.2)	3 (3.4)	10 (4.5)	17 (5.1)
Rash^d^	7 (2.9)	6 (3.4)	13 (3.1)	17 (3.2)	4 (3.0)	0	4 (1.8)	2 (0.6)
Mental impairment^e^	5 (2.1)	2 (1.1)	7 (1.7)	12 (2.2)	1 (0.7)	1 (1.1)	2 (0.9)	8 (2.4)
Local AEs (> 5% of patients in any subgroup)								
Diarrhea	22 (9.2)	17 (9.6)	39 (9.4)	32 (5.9)	12 (9.0)	7 (7.9)	19 (8.5)	12 (3.6)
Constipation	14 (5.9)	13 (7.3)	27 (6.5)	39 (7.2)	10 (7.5)	11 (12.4)	21 (9.4)	15 (4.5)
Abnormally frequent urination^f^	15 (6.3)	11 (6.2)	26 (6.3)	16 (3.0)	4 (3.0)	4 (4.5)	8 (3.6)	10 (3.0)
Hematuria	14 (5.9)	6 (3.4)	20 (4.8)	23 (4.3)	4 (3.0)	6 (3.4)	13 (5.8)	17 (5.1)
Dysuria	2 (0.8)	9 (5.1)	11 (2.7)	14 (2.6)	2 (1.5)	3 (3.4)	5 (2.2)	24 (7.3)

Abbreviations: AE, adverse event; RP, prostatectomy; RT, radiotherapy.

^a^ One patient had no information on prior local therapy.

^b^ One randomized patient did not receive study treatment and was excluded from safety analyses.

^c^ Includes Medical Dictionary for Regulatory Activities (MedDRA) preferred terms: any fractures and dislocation, limb fractures and dislocations, skull fractures, facial bone fractures and dislocations, spinal fractures and dislocations, and thoracic cage fractures and dislocations.

^d^ Includes MedDRA preferred terms: rash, macular rash, maculopapular rash, papular rash, and pustular rash.

^e^ MedDRA high‐level group term.

^f^ Pollakiuria.

The incidence of AEs commonly associated with ARIs was generally similar across the prior therapy subgroups and between the darolutamide and placebo groups (Table 3). Consistent with the overall population, fatigue was the most reported AE with darolutamide among the prior therapy subgroups (range, 11.3%–17.6%). Differences in AE incidences between darolutamide and placebo groups for the AEs associated with ARIs were < 4.5% for the prior therapy subgroups, except for fatigue among those who received prior therapy. Incidences of local AEs that may be related to local therapy were infrequent and similar across prior therapy subgroups. The most commonly reported local AEs (> 5% of patients in any subgroup) were diarrhea, constipation, abnormally frequent urination, hematuria, and dysuria.

## 4 Discussion

In this post hoc analysis of ARAMIS, efficacy, safety, and HRQoL outcomes associated with darolutamide were evaluated based on the use of prior local prostate‐directed therapy. In terms of efficacy, we found that patients with nmCRPC benefited from darolutamide, with a median MFS exceeding 40 months independent of prior local therapy. Darolutamide showed an OS benefit across prior local therapy subgroups, with a more pronounced effect versus placebo in patients without prior RP or RT. Despite similar patient characteristics at study entry, patients without prior local therapy progress more quickly, and the OS benefit with darolutamide versus placebo was greater in this subgroup. This finding highlights the benefit of darolutamide plus ADT for a group of patients who may not have been suitable for prior local therapy for reasons that might include older age, presence of comorbidities, worse performance status, or worse disease characteristics. Exposure to prior local therapy is associated with better prognosis [12, 14], and in our analysis, patients with prior RP had the best 3‐year survival rate of 89.6%. Furthermore, darolutamide treatment showed a benefit over placebo in delaying the time to PSA progression and increasing PSA50 response regardless of prior local therapy and consistent with the overall ARAMIS population [7]. Interaction analyses for MFS, OS, and time to PSA progression showed no interaction between treatment groups and prior local therapy, indicating that darolutamide benefits patients with or without prior local therapy.

In a previous analysis of the ARAMIS trial, darolutamide led to a statistically significant delay in deterioration of EORTC QLQ‐PR25 urinary and bowel subscales and FACT‐P PCS versus placebo [19, 20]. In the current analysis, patients receiving darolutamide had longer median DetFS of HRQoL on the EORTC QLQ‐PR 25 urinary and bowel subscales regardless of prior therapy. The treatment effect was more pronounced in patients who received prior RP or RT compared with patients who did not receive RP or RT and is consistent with the effect of darolutamide on MFS. Patients in the placebo group who did not receive prior local therapy had longer times to EORTC QLQ‐PR 25 urinary symptom DetFS versus those who received prior RP or RT, suggesting that prior local therapy may have a negative impact on urinary symptom‐related HRQoL while showing slower disease progression as observed by higher 3‐year OS rates compared with no prior RP or RT. Median DetFS times for EORTC QLQ‐PR 25 bowel symptoms were similar for patients in the placebo group with and without prior local therapy, suggesting minimal negative effects of these local treatments on bowel symptoms. For the FACT‐P PCS, which measures both general and prostate cancer–specific HRQoL, darolutamide improved DetFS versus placebo, with a greater treatment effect in patients who received prior RP or RT versus those who did not receive prior RP or RT.

Local therapy is associated with long‐term gastrointestinal, sexual, and urinary AEs [21]. The safety and tolerability profile of darolutamide was similar across the prior therapy subgroups and consistent with the ARAMIS population in the primary and extended follow‐up analyses [7, 8]. Darolutamide has a distinct molecular structure from other approved ARIs that strongly inhibits androgen receptor binding and has low penetration of the blood–brain barrier [6]. Treatment with other ARIs has been associated with a higher incidence of central nervous system–related AEs, including falls and mental impairment disorders, as well as fatigue, rash, and hypertension [22, 23]. The darolutamide group had a ≤ 2% difference compared with placebo for most AEs commonly associated with ARIs (i.e., falls, fractures, mental impairment, rash, and hypertension) [7, 8, 22, 23]. Fatigue was the only AE with an incidence > 10%, occurring in 13.2% of patients receiving darolutamide versus 8.3% of patients receiving placebo [8]. Except for fatigue, the difference in AEs commonly associated with ARIs was < 4.5% between darolutamide and placebo prior local therapy subgroups, which is similar to the overall ARAMIS population considering the smaller subgroup populations [8]. The incidence of local AEs that may be related to local prior therapy, such as diarrhea, constipation, abnormally frequent urination, hematuria, and dysuria, was infrequent and similar across prior therapy subgroups. The discontinuation rates due to AEs in the darolutamide group were not affected by prior treatments and remained consistently low and similar to those of the placebo group [8].

The findings of this post hoc analysis are consistent with those from the overall population of the long‐term ARAMIS trial [7, 8]. Use of prior local therapy was prospectively collected in ARAMIS, but this subset analysis was not prespecified and is limited by the small size of subgroups. Therefore, this analysis has several limitations and only inferences can be made about the individual subgroups. There were some differences in the baseline characteristics across the subgroups, such as a shorter time from diagnosis and a higher percentage of patients with an ECOG performance status of 1 in the prior local therapy group, which may confound some findings. Additional differences may not be evident due to other confounders not measured in this analysis. During the open‐label period of ARAMIS, the darolutamide group continued treatment and 170 patients previously receiving placebo were allowed to receive darolutamide, which may have impacted outcomes in the placebo group [7]. Additionally, post hoc analyses may be inherently susceptible to bias, and the results here should be confirmed by well‐designed prospective studies that consider use of prostate‐specific membrane antigen positron emission tomography, or PSMA PET, for cancer staging and earlier use of intensified treatments. Interestingly, a notable proportion of patients with nmCRPC included in the ARAMIS study received no prior local therapy (56% for darolutamide and 60% for placebo). This observation is consistent with real‐world patterns and may be attributed to a number of factors, which include presentation of advanced disease at diagnosis, older age and presence of comorbidities, patient preference, and quality of life considerations [24, 25, 26, 27]. Understanding the effects of prior local therapy on later outcomes, such as failure of systemic therapy, is interesting and warrants further study.

## 5 Conclusions

In this post hoc analysis, patients with nmCRPC benefited from darolutamide independent of prior local therapies to treat the primary tumor, with consistent effects on disease progression and survival as observed in the overall ARAMIS population. Darolutamide also delayed time to deterioration in HRQoL, including urinary and bowel symptoms. The improved disease control compared with placebo and low incidence of AEs support the benefits of darolutamide for patients with and without prior RP or RT.

## Author Contributions

**Matthias Saar:** conceptualization, investigation, resources, writing – review and editing. **Karim Fizazi:** conceptualization, investigation, resources, writing – review and editing. **Neal D. Shore:** conceptualization, investigation, resources, writing – review and editing. **Matthew Smith:** conceptualization, investigation, resources, writing – review and editing. **Jan‐Erik Damber:** investigation, resources, writing – review and editing. **Andrey Semenov:** investigation, resources, writing – review and editing. **Maria J. Ribal:** investigation, resources, writing – review and editing. **Alison Birtle:** investigation, resources, writing – review and editing. **Jérôme Rigaud:** investigation, resources, writing – review and editing. **Christopher J. D. Wallis:** conceptualization, investigation, resources, writing – review and editing. **Marc‐Oliver Grimm:** investigation, resources, writing – review and editing. **Susan Halabi:** investigation, resources, writing – review and editing. **Andrew J. Armstrong:** investigation, resources, writing – review and editing. **Ateesha F. Mohamed:** conceptualization, methodology, formal analysis, writing – review and editing. **Patrick Adorjan:** conceptualization, writing – review and editing. **Shankar Srinivasan:** conceptualization, methodology, formal analysis, writing – review and editing. **Frank Verholen:** conceptualization, methodology, writing – review and editing. **Alicia K. Morgans:** conceptualization, investigation, resources, writing – review and editing. **D. Robert Siemens:** conceptualization, investigation, resources, writing – review and editing.

## Ethics Statement

Per the primary publication of the ARAMIS study (Fizazi et al. *N Engl J Med*. 2019;380:1235–1246), the institutional review board at each participating institution approved the trial, which was conducted in compliance with the principles of the Declaration of Helsinki and in accordance with the International Conference on Harmonisation guidelines for Good Clinical Practice. All patients provided written informed consent.

## Conflicts of Interest

Matthias Saar: Consulting or Advisory Roles: Accord, Amgen, Astellas, AstraZeneca, Bayer, Bristol Myers Squibb, Johnson & Johnson, Ipsen, Merck, Novartis, Pfizer, Merck Sharp & Dohme; Travel, Accommodation, and Expenses: Johnson & Johnson, Bayer, Merck Sharp & Dohme; Grant (to institution): Novartis. Karim Fizazi: Consulting or Advisory Roles with honoraria (to institution): Amgen, Astellas, AstraZeneca, Bayer, Clovis, Daiichi Sankyo, Janssen, Merck Sharp & Dohme, Novartis/AAA, Pfizer, Sanofi; Advisory Roles with personal honoraria: Arvinas, CureVac, Macrogenics, Orion. Neal D. Shore: Consulting or Advisory Role: Bayer, Janssen Scientific Affairs, Dendreon, Tolmar, Ferring, Medivation/Astellas, Amgen, Pfizer, AstraZeneca, Myovant Sciences, Astellas Pharma, AbbVie, Merck, Bristol Myers Squibb/Sanofi, Boston Scientific, Clovis Oncology, Exact Imaging, FerGene, Foundation Medicine, CG Oncology, InVitae, MDxHealth, Myriad Genetics, Nymox, Propella Therapeutics, Genzyme, Sanofi, Sesen Bio, CG Oncology, Exact Sciences, Genesis Cancer Care, Pacific Edge Biotechnology, Phosphorus, Urogen Pharma, Speciality Networks, Peerview, Clarity Pharmaceuticals, Lantheus, Lilly, Photocure, Sema4, Telix Pharmaceuticals, Tempus, Vaxiion; Speakers' Bureau: Janssen, Bayer, Dendreon, Astellas Pharma, AstraZeneca, Clovis Oncology, Pfizer, Guardant Health, Merck, Foundation Medicine; Research Funding: AbbVie, Amgen, Astellas Pharma, AstraZeneca, Bayer, Bristol Myers Squibb/Pfizer, Boston Scientific, Clovis Oncology, Dendreon, Exact Imaging, Ferring, Foundation Medicine, InVitae, Janssen, MDxHealth, Merck, Myovant Sciences, Nymox, Pfizer, Sanofi, Sesen Bio, Tolmar, CG Oncology, DisperSol, FORMA Therapeutics, Guardant Health, Jiangsu Yahong Meditech, Novartis, Pacific Edge, POINT Biopharma, Propella Therapeutics, Seattle Genetics, MT Group, Theralase, Veru, Zenflow, Advantagene, Aragon Pharmaceuticals, Endocyte, Exelixis, FKD Therapies, Genentech, Istari Oncology, Medivation, OncoCellMDx, ORIC Pharmaceuticals, Palette Life Sciences, Plexxikon, RhoVac, Steba Biotech, Urogen Pharma, Urotronic, US Biotest, Vaxiion; Expert Testimony: Ferring; Other Relationship: Photocure, Alessa Therapeutics. Matthew Smith: Consulting or Advisory Role: Bayer, Janssen Oncology, Amgen, Pfizer, Lilly, Novartis, Astellas Pharma; Research Funding (to institution): Janssen Oncology, Bayer, Lilly, ESSA, ORIC Pharmaceuticals. Jan‐Erik Damber: Nothing to disclose. Andrey Semenov: Research Funding: Astellas, Janssen, AstraZeneca, Merck Sharp & Dohme, Hoffmann La‐Roche. Maria J. Ribal: Research Funding: Bayer, Janssen, Unicancer, Astellas, Seagen, Urogen Pharma, Fidia, DMIA; Honoraria: Bayer, Johnson & Johnson; Patents: METODO DE DIAGNOSTICO NO INVASIVO DE CANCER DE VEJIGA. Inventores: Antonio Alcaraz, Lourdes Mengual, María José Ribal, Juan José Lozano. Oficina de patente: European Patent Office. Número de concesión: 13382030.8‐1403. Entidad titular: Fina Biotech, S.L.U. Junio 2007. Alison Birtle: Consulting or Advisory Role: Astellas Medivation, AstraZeneca, Bayer Schering Pharma, Bristol Myers Squibb, Gilead, Janssen, Merck, Macrogenics, Merck Sharpe & Dohme, Pfizer, Roche, Sanofi; Honoraria: Janssen; Speakers' Bureau: Bayer, Janssen Oncology, Astellas, Merck, Pfizer; Research Funding: Genzyme. Jérôme Rigaud: Nothing to disclose. Christopher J. D. Wallis: Consulting Fees: Janssen Oncology, Nanostics Precision Health, Precision Point Specialty LLC, SESEN Bio; Honoraria/Travel: AbbVie, Astellas, Astra Zeneca, Bayer, EMD Serono, Haymarket Media, Healing and Cancer Foundation, Knight Therapeutics, Intuitive Surgical, Merck, Science & Medicine Canada, Sumitomo Pharmaceuticals, TerSera Canada, Tolmar Pharmaceuticals Canada; Research Funding: Knight Therapeutics, Tolmar Pharmaceuticals, Bayer. Marc‐Oliver Grimm: Consulting or Advisory Role: AstraZeneca, Bristol Myers Squibb, Ipsen, Merck Sharp & Dohme, Pfizer, Astellas, EUSA Pharma, Merck Serono, Roche Pharma AG, Eisai, Bayer Vital, Janssen, Gilead, Novartis, Takeda; Grants: Bristol Myers Squibb, Intuitive Surgical, Bayer Vital. Susan Halabi: Employment: ASCO, Honoraria for Data Monitoring Committee: AVEO, Bristol Myers Squibb, BeiGene, CG Oncology, Janssen, Sanofi. Andrew J. Armstrong: Consulting or Advisory Role: Astellas Scientific and Medical Affairs Inc., AstraZeneca, Bayer, Bristol Myers Squibb, Epic Sciences, Exelixis, FORMA Therapeutics, GoodRx, IDEAYA Biosciences, Janssen, Merck, Myovant Sciences, Novartis, Pfizer, Research Funding (to institution): Amgen, Astellas Pharma, AstraZeneca, Bayer, BeiGene, Bristol Myers Squibb, Constellation Pharmaceuticals, Dendreon, FORMA Therapeutics, Gilead Sciences, Janssen Oncology, Merck, Novartis, Pfizer; Patents, Royalties, Other Intellectual Property (to institution): Circulating tumor cell novel capture technology; Travel, Accommodations, Expenses: Astellas Scientific and Medical Affairs Inc. Alicia K. Morgans: Honoraria: AstraZeneca, Astellas, Bayer, Curium, Exelixis, Janssen, Lantheus, Merck, Novartis, Pfizer, Sumitomo Pharma Inc., Telix, Tolmar; Research Funding: Astellas, Bayer, Janssen, Pfizer, Sumitomo Pharma Inc. D. Robert Siemens: Research Funding of Clinical Trials: Bayer, AstraZeneca, Merck Sharpe & Dohme, Pfizer; Employment: American Urological Association (AUA) as editor‐in‐chief of *The Journal of Urology*. Ateesha F. Mohamed, Patrick Adorjan, Shankar Srinivasan, and Frank Verholen: employees of Bayer.

## Supporting information


Appendix S1:cam471343‐sup‐0001‐AppendixS1.docx.

## Data Availability

Availability of the data underlying this publication will be determined according to Bayer's commitment to the EFPIA/PhRMA “Principles for responsible clinical trial data sharing”. This pertains to scope, timepoint, and process of data access. As such, Bayer commits to sharing upon request from qualified scientific and medical researchers patient‐level clinical trial data, study‐level clinical trial data, and protocols from clinical trials in patients for medicines and indications approved in the United States (US) and European Union (EU) as necessary for conducting legitimate research. This applies to data on new medicines and indications that have been approved by the EU and US regulatory agencies on or after January 01, 2014. Interested researchers can use www.vivli.org to request access to anonymized patient‐level data and supporting documents from clinical studies to conduct further research that can help advance medical science or improve patient care. Information on the Bayer criteria for listing studies and other relevant information is provided in the member section of the portal. Data access will be granted to anonymized patient‐level data, protocols, and clinical study reports after approval by an independent scientific review panel. Bayer is not involved in the decisions made by the independent review panel. Bayer will take all necessary measures to ensure that patient privacy is safeguarded.
