# Supplementary material for: Effects of Prior Local Therapy by Radical Prostatectomy or Radiotherapy on the Efficacy and Quality of Life of Patients Treated With Darolutamide in ARAMIS
Source: Cancer Med. 2025 Dec 26;15(1):e71343. doi: 10.1002/cam4.71343 (PMC12741647; doi:10.1002/cam4.71343)

**SUPPORTING INFORMATION**

**TABLE S1** | Metastasis-free survival rate for the darolutamide group by prior local therapy.

|  | **Darolutamide^a^** | | | | |
| --- | --- | --- | --- | --- | --- |
|  | **RP**  **(*n* = 239)** | **RT**  **(*n* = 177)** | **RP or RT**  **(*n* = 416)** | **Neither RP nor RT**  **(*n* = 538)** | **All**  **(*n* = 954)** |
| Metastasis-free survival, median (95% CI), months | 40.4  (30.8–NE) | NE  (33.3–NE) | 40.4  (32.9–NE) | 40.5  (35.8–NE) | 40.4  (34.3–NE) |
|  | **Placebo^a^** | | | | |
|  | **RP**  **(*n* = 134)** | **RT**  **(*n* = 89)** | **RP or RT**  **(*n* = 223)** | **Neither RP nor RT**  **(*n* = 331)** | **All**  **(*N* = 554)** |
| Metastasis-free survival, median (95% CI), months | 14.7  (11.1–32.8) | 18.4  (14.6–22.4) | 14.8  (14.5–22.3) | 19.1  (18.3–29.2) | 18.4  (15.5–22.3) |

Abbreviations: NE, not estimated; RP, radical prostatectomy; RT, radiotherapy.
^a^During central review of conventional imaging, 50 (5.2%) patients in the darolutamide group and 29 (7.0%) patients in the placebo
group were found to have metastasis at baseline. One patient had no information on prior local therapy.

**TABLE S2** | Relative contribution of MFS, HRQoL deterioration, and treatment discontinuation events to total events for DetFS endpoint for each HRQoL measure by treatment group.

|  | Darolutamide (*N* = 954) | | | |  | Placebo (*N* = 554) | | | |
| --- | --- | --- | --- | --- | --- | --- | --- | --- | --- |
| **HRQoL DetFS measure** | **Total Events** | **MFS, *n* (%)** | **HRQoL deterioration, *n* (%)** | **Treatment DC, *n* (%)** |  | **Total Events** | **MFS, *n* (%)** | **HRQoL deterioration, *n* (%)** | **Treatment DC, *n* (%)** |
| EORTC QLQ-PR25 | | | | | | | | | |
| Urinary | 476 | 142 (29.8) | 266 (55.9) | 68 (14.3) |  | 320 | 145 (45.3) | 144 (45.0) | 31 (9.7) |
| Bowel | 532 | 134 (25.2) | 328 (61.7) | 70 (13.2) |  | 322 | 141 (43.8) | 157 (48.8) | 24 (7.5) |
| FACT-P |  |  |  |  |  |  |  |  |  |
| PCS | 498 | 130 (26.1) | 301 (60.4) | 67 (13.5) |  | 311 | 155 (49.8) | 125 (40.2) | 31 (10.0) |

Abbreviations: DC, discontinuation; EORTC QLQ-PR25, European Organisation for Research and Treatment of Cancer Quality of Life Questionnaire Prostate Cancer Module; FACT-P, Functional Assessment of Cancer Therapy-Prostate; HRQoL, health-related quality of life; MFS, metastasis-free survival; PCS, prostate cancer subscale.

**FIGURE S1** | Overall 3-year survival rate (95% CI) by prior therapy among the (A) darolutamide group and (B) placebo group. RP, radical prostatectomy; RT, radiotherapy.

**(A)**


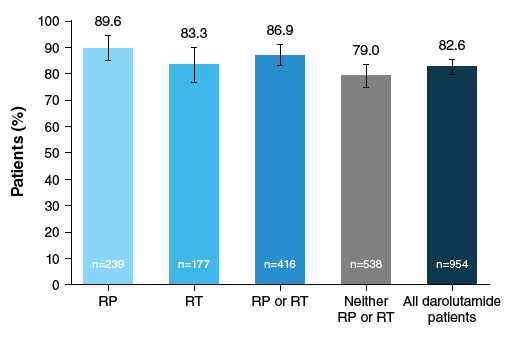


**(B)**


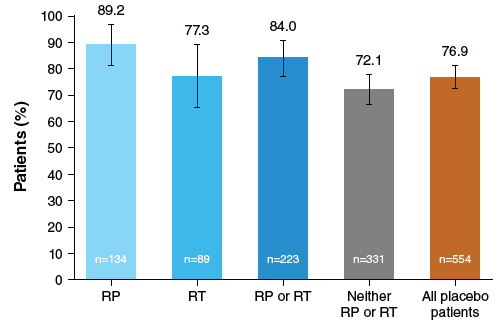


**FIGURE S2** | PSA50 response rate (95% CI) by prior therapy for the (A) darolutamide group and (B) placebo group. RP, radical prostatectomy; RT, radiotherapy.

**(A)**


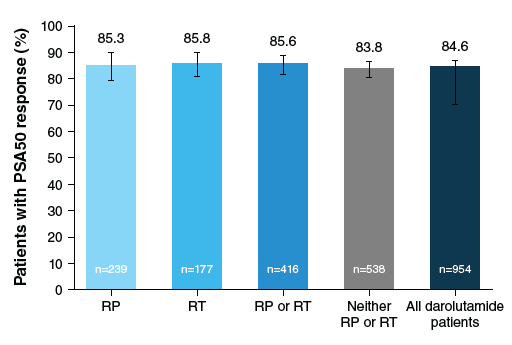


**(B)**


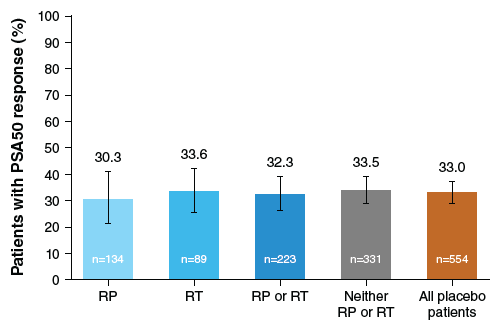

Supplement: Supplementary file 1 — Appendix S1:cam471343‐sup‐0001‐AppendixS1.docx. [file CAM4-15-e71343-s001.docx]
